# Ruminal impaction due to plastic materials - An increasing threat to ruminants and its impact on human health in developing countries

**DOI:** 10.14202/vetworld.2018.1307-1315

**Published:** 2018-09-20

**Authors:** M. Priyanka, S. Dey

**Affiliations:** 1 Animal Experimentation Station, Indian Veterinary Research Institute, Yelahanka, Bengaluru, Karnataka, India; 2 Division of Medicine, Indian Veterinary Research Institute, Izatnagar, Bareilly, Uttar Pradesh, India

**Keywords:** developing countries, human health, plastic materials, ruminal impaction, ruminants, urban areas

## Abstract

Ruminal impaction due to plastic materials is a condition, in which indigestible plastic foreign bodies accumulate in the rumen of ruminants leading to ruminal impaction, indigestion, recurrent tympany, and many other adverse health effects. It is caused by the indiscriminate feeding of ruminants on indigestible plastic waste materials. The disease is primarily noticed in stray animals residing in urban areas of developing countries. Ingested plastic materials in the rumen slowly release the chemicals in rumen fluid, which intern enter the food chain through milk and meat products. These chemicals have a detrimental effect on human health. At present, exploratory rumenotomy is the only choice for both diagnosis and treatment of ruminal impaction due to plastic materials in ruminants. Control measures include good animal husbandry practices and proper disposal of plastic waste materials. The present review discusses in depth about the epidemiology, pathophysiology, diagnosis, treatment, prevention, and control of ruminal impaction due to plastic materials in ruminants and also highlights its impact on human health.

## Introduction

Plastic (from Greek word *plastikos* meaning moldable) popularly known as the materials for the 21^st^ century, is a synthetic or semi-synthetic organopolymeric compound that can be molded into the solid substance of any shape. Alexander Parkes invented plastics in the 1860s, and its industrial application was discovered, in 1920s, by Fenichell [1]. Since then plastic production is getting exploded and plastic industry has become one of the fastest growing global industries [2]. At present, global plastic production is about 322 million metric tons, and the global plastic industry generates revenue of about $600 billion annually [3,4]. Increasing population and the growth of manufacturing sectors in developing countries have increased the demand for plastic production [2]. In recent years’ plastic production is also shifted to Asian countries. Developing Asian countries such as China and India are contributing about 34.8% of the world’s plastic production [2,3]. Due to low cost, ease of production, versatility and moisture resistance, and plastics have gained a lot of industrial applications and have already displaced many traditional materials such as wood, stone, leather, and many others.

These are used in manufacturing an enormous and expanding range of products, from paper clips to spaceships [5]. Globally, there is an exponential increase in the per capita consumption of plastics. In the 1980’s world average per capita plastic consumption was about 11 kg per person, and, in 2015, it is about 45 kg per person [4]. Studies have estimated that in the next 5 years, modernization and rising consumerism will double the per capita utilization of plastics especially in urban areas of developing countries [6]. Consumption pattern of plastics is different in both developing and developed countries [2]. In developed countries, construction and automobiles consume about 53% of plastics, and around 33% of plastics are used in packaging food and beverages [5]. Whereas, in developing countries, around 44-48% of plastics are used in packaging food and beverages [5].

In proportion to the growth of plastic industry, generation of plastic waste is also increasing. However, recovery and recycling of this waste remain insufficient leading to the accumulation of these plastics in landfills and oceans every year [7]. Landfilling is the first option in many countries. Between 22% and 43% of plastic worldwide is disposed of in landfills [3]. Western countries such as US, UK, and Germany are handling their plastic waste through well-established waste collection systems, recycling, and exporting to other countries. China receives about 56% of plastic waste worldwide, making it the world’s largest importer of plastic waste [3]. Handling of plastic waste is improper in developing countries because of low environmental standards, poor waste recovery and disposal systems, low economic status, poor hygienic and living standards, less awareness of public regarding harmful effect of plastics, no stringent/strict law regarding waste disposal, and many other factors [7]. All this leads to accumulation of plastic waste on roadsides and open grounds. The United Nations Environment Programme, 2016, estimates that 57% of the plastic in Africa, 40% in Asia, and 32% in Latin America are not even collected, being instead littered or burned in the open. China, Sri Lanka, Egypt, Nigeria, Bangladesh, South Africa, Indonesia, the Philippines, Thailand, Vietnam, and India are the major countries contributing to massive environmental pollution through dumping plastic waste in landfills and oceans every year [8].

In most of the developing countries, especially in urban areas, animals are left to graze freely in open areas [9]. Animals in these areas graze on plastic garbage leading to the ingestion of plastic waste materials and development of ruminal impaction due to plastic materials [10]. Ruminal impaction due to plastic materials is a condition, in which indigestible plastic foreign bodies accumulate in the rumen leading to ruminal impaction, indigestion, recurrent tympany, and death. The present paper describes in depth about ruminal impaction due to plastic materials in ruminants and its possible impact on human health in developing countries.

## Etiology

Animals ingest these plastic materials due to erratic feeding behavior, confusing for food and when trying to eat leftover feed materials in plastic wrappings [11]. Ingestion of these indigestible plastic materials over a period of time leads to the development of ruminal impaction due to plastic materials in ruminants [12].

## Predisposing Factors

There are several factors which play an important role in predisposing the animals to the ingestion of plastic waste.

### Type of grazing system

Livestock reared under the extensive type of farming is more susceptible to the development of ruminal impaction due to plastic materials [11,13]. As these animals are left free for grazing, they are at risk of feeding on plastic waste. Animals maintained under the intensive farming system are not exposed to waste. Hence, these animals rarely develop plastic foreign body syndrome.

### Mineral deficiencies

Calcium and phosphorous deficiency will cause capricious appetite in animals. To satisfy their hunger, animals start eating inanimate objects and lead to the development of foreign body syndrome [11,14].

### Negative energy balance

Poor nutritional supplementation and increased energy requirement during pregnancy and lactation make the animals to be in negative energy balance status. To meet the energy requirements, animals consume inanimate objects and develop the foreign body syndrome [11,15].

### Urbanization

Rapid urbanization has diminished the resources and grazing lands of animals. In addition, extensive construction works are carried out in urban areas, and proper disposal of plastic waste is not being followed. Waste is thrown anywhere on roads and near fences, making it easy for animals to graze on them and develop ruminal impaction due to plastic materials [14]. Therefore, this condition is more prevalent in urban and peri-urban areas [15].

### Ban on slaughter of certain animals

Slaughter of certain types of animals is banned in some countries. Due to the low economy, livestock owners will abandon unproductive animals. In search of food, livestock will graze on streets, roadsides, waste dumping yards, and polluted areas leading to the development of ruminal impaction due to plastic materials [14,15].

### Industrialization

In recent years, there is rapid growth in plastic industries in developing Asian countries [2]. It had increased the consumption of plastic and production of plastic waste. The ineffective waste collection, improper waste disposal, and poor standards of animal rearing have increased the incidence of ruminal impaction due to plastic materials in developing countries.

### Economic status of the country

Ruminal impaction due to plastic materials is common in the animals residing in countries of low economic status. An ineffective waste disposal system, poor standards of living, less awareness about the harmful effects of plastic waste, and poor animal husbandry practices in these countries predispose them to the ruminal impaction due to plastic materials [2].

### Draught and flood

Ruminal impaction due to plastic materials is common in drought and flood-prone areas. Long periods of drought and floods will cause a fodder shortage in these areas. No prior preparedness to such situations which affect fodder availability to livestock. This, in turn, causes the indiscriminate grazing and occurrence of foreign body syndrome in livestock [15].

### Type of plastic materials

In general, ruminal impaction due to plastic materials is caused by ingestion of polythene bags, plastic covers, and other plastic materials used in the packaging of food products [11,16,17].

## Epidemiology of Ruminal Impaction due to Plastic Materials

### Prevalence

Ruminal impaction due to plastic materials in ruminants has been reported in many countries. Plastic bags are the most common type of foreign bodies found in ruminants suffering from impaction. Slaughterhouse prevalence of plastics among other foreign bodies in ruminal impaction has been reported by different workers all over the world. Hailat *et al*. reported a 74% prevalence in Jordan [18]; Ruminal impaction due to plastic materials in ruminants has been reported in many countries worldwide. Plastic bags are the most common type of foreign bodies found in ruminants suffering from impaction. Slaughterhouse prevalence of plastics among other foreign bodies in ruminal impaction has been reported by different workers all over the world. In Jordan slaughterhouse prevalence was found to be around 74 % [18]; in Pakistan it was about 62.5% [10]; in Nigeria it was reported to be 81.6% and 85% by different workers [16,19]; in Ethiopia it was 50% [15]; in Kenya it was 72.3% [20]; and 79.2% prevalence in eastern Ethiopia [21]. Although studies indicating that the prevalence of ruminal impaction due to plastic materials in ruminants is lacking from a few countries such as India, Egypt, Yemen, and others, many workers have reported the case studies regarding the occurrence of ruminal impaction due to plastic materials in ruminants from these countries [13, 22-25].

### Susceptibility

#### Species

Ruminal impaction due to plastic materials is observed in all domestic and wild ruminants [17]. Among domestic ruminants, cattle are more susceptible to the development of ruminal impaction due to plastic materials followed by buffalo, sheep, and goat. This can be attributed to the prehensile nature of these animals. Bovine species does not have highly sensitive prehensile organs such as lips and tongues that discriminate sense of taste and making them indiscriminate feeders. Bovines also graze close to the ground making them more vulnerable to ingestion of plastic foreign bodies. Caprine are less susceptible to the ingestion of foreign bodies because of their well-developed sensitive prehensile organs and browsing and selective feeding behavior [26]. Fodder scarcity, environmental contamination, and poor standards of animal rearing are forcing goats to ingest and accumulate plastic foreign bodies in their rumen [15]. Cases of plastic foreign body impaction had been reported in wild ruminants due to the contamination of their habitat by plastic waste [27].

#### Breed

Crossbreed cattle are more susceptible than local cattle to the development of ruminal impaction due to plastic materials [15]. As crossbreed cattle have more requirements for feed and fodder, it predisposes them for the ingestion of foreign bodies.

#### Sex

Sex of the animal strongly influences the ingestion of foreign bodies in animals. Female animals are more susceptible to the development of ruminal impaction due to plastic materials than male animals [14,16]. In female animals, various physiological factors contribute to the ingestion of foreign bodies. Greater nutritional demand, negative energy balance, and mineral deficiency during pregnancy and lactation stages increase the appetite of these animals leading to consumption of foreign bodies [11,18]. Farmers keep female animals longer than male animals because of their long reproductive period. Therefore, female animals are at higher risk of exposure through life to ingestion and accumulation of foreign bodies in rumen [11]. Abandoning of unproductive female animals and ban of slaughter of female animals like cow in countries like India have compelled them to graze on roadsides and waste disposal areas leading to the ingestion of foreign bodies.

#### Age

Old animals are more susceptible to the development of ruminal impaction due to plastic materials than young animals [15]. Cattle above 10 years of age and sheep and goat above 4 years of age were more frequently affected with indigestible materials than the other age group [28-30]. This might be associated with an increase in exposure through life and animals will ingest and accumulate foreign bodies gradually over a period of time [15]. Aged animals are culled due to low productivity and poor economic returns. These animals end up surviving on garbage leading to the ingestion of foreign bodies.

#### Body condition score

Animals with poor body condition are the most affected groups compared to that of good body condition [14,15]. Poor nutritional status and negative energy balance cause capricious appetite in animals leading to the ingestion of indigestible food materials and development of ruminal impaction due to plastic materials.

#### Season

The incidence of ruminal impaction due to plastic materials is noticed during dry period, i.e., between March and July. In dry period, fodder scarcity is the major problem in the areas of dryland and rainfed agriculture. In dry season, animals will roam from one place to the other in search of feed and fodder. This makes them prone to the ingestion of foreign bodies. During lean seasons of agriculture, livestock owners along with their animals will migrate to urban areas in search of job. In urban areas, they will leave their livestock freely to find their food. Due to non-availability of feed and indiscriminate disposal of polythene materials with some food remnants on, livestock will graze on this waste leading to ingestion of plastic bags along [11,14].

### Type of occurrence

Most of the cases of ruminal impaction due to plastic materials occur sporadically in and around urban areas.

## Economic Loss

Ruminal impaction due to plastic materials causes economic loss to farmers in terms of low milk yield, poor weight gain, reduced draft ability, other comorbid disease conditions, and mortality. In Jordan, an estimated loss of 15 million USD in productivity and health was associated with plastic impaction in sheep [31].

## Pathogenesis

**Figure-1:**
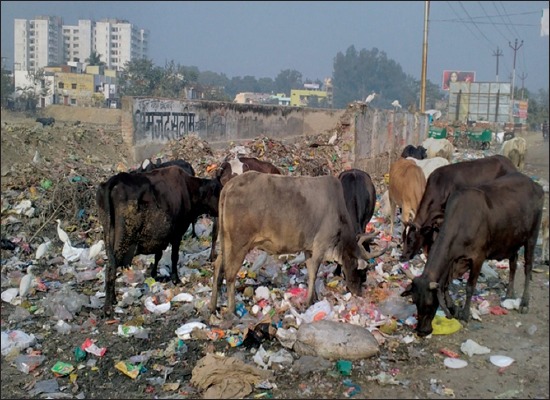
Grazing of cattle on plastic waste materials.

**Figure-2:**
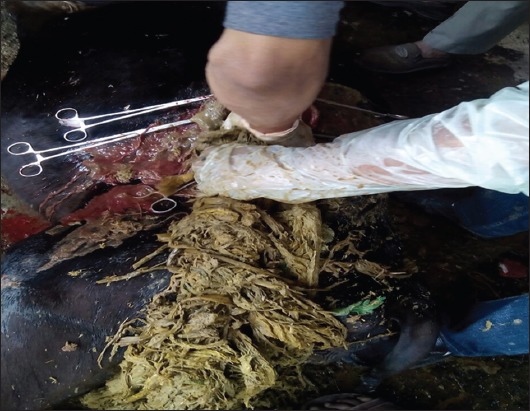
Rumen impacted by plastic waste materials.

**Figure-3:**
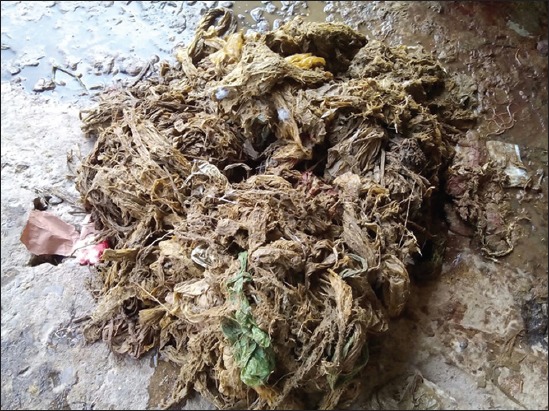
Impacted plastic waste materials in Rumen.

**Figure-4:**
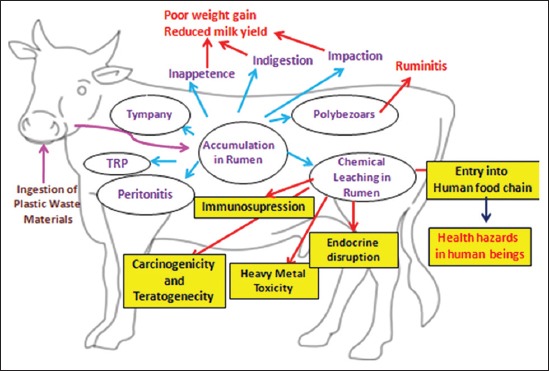
Pathophysiology of ruminal impaction due to plastic materials in ruminants (Conditions mentioned in yellow box indicate the probable outcome after chronic exposure to the products of chemical leaching in rumen. However no studies are conducted in this direction). [Figure designed by M. Priyanka].

In confusion for food, animals will feed on plastic waste materials such as polybags and plastic covers (Figure-1) [17]. As these plastic materials are indigestible, they are lodged in the rumen and then move to reticulum and omasum (Figures-2 and 3) [32]. Depending on the type and amount of plastic waste ingested, type of material in plastic waste, duration of plastic waste accumulated in forestomach, and location of this plastic foreign body in gastrointestinal tract, various pathological conditions are encountered in animals. Singh [33] stated that indigestion, impaction, tympany, polybezoars, traumatic reticulopericardtis, chemical leaching and immunosuppression are the pathological conditions encountered in animals with ruminal impaction due to plastic materials. Apart from these, there is possibility of occurrence of certain other conditions such as heavy metal toxicities, endocrine disruption, carcinogenicity, teratogenicity, and urolithiasis due to ruminal impaction with plastic materials in ruminants. However, till today these conditions are not reported. All the associated conditions of ruminal impaction due to plastic materials are illustrated in Figure-4.

## Inappetence

The polythenes and other plastic material do not degrade in rumen/reticulum and remain as such. Their physical presence in rumen will stretch the cranial sac of the rumen. It will stimulate the ventromedial hypothalamus and satiety center leading to loss of appetite [32,34].

## Simple Indigestion

The plastic bags and other plastic material cannot be digested or passed as such through feces by an animal [34]. Their continuous presence in rumen will cause atrophy of ruminal papillae and thereby affect the normal digestion and fermentation process [18,35]. Due to the churning action of ruminal contractions, plastics get entangled with each other. In the rumen, feed ingredients get trapped in between the plastic materials and become unavailable to rumen protozoa for the digestive and fermentative process. This affects the rumen microflora function leading to indigestion [33].

## Ruminal Impaction

Initially, due to ruminal contractions, polythene bags/plastics accumulated in rumen will get entangled with each other leading to the formation of hard mass. Later on, this hard plastic mass obstructs the orifice between reticulum and omasum thereby causing hindrance to the ruminal movements. Over a period of time, this hard plastic mass leads to decrease in rumen motility and thereby cause ruminal atony and ruminal impaction [33,36]. Ruminal impaction by plastic foreign body is asymptomatic and is diagnosed only after the accumulation of huge quantities of plastic materials in rumen [37].

## Ruminal Tympany

Polythenes present in rumen and reticulum will partially or completely occlude the orifice of reticulum and omasum leading to the accumulation of gases in rumen [16,19]. The situation worsens if such animals are fed with legumes or other gas forming feed/concentrates. Accumulation of gases in rumen gives rise to bloat or tympany which becomes fatal if the gases are not properly removed. Sometimes the poly bags present in rumen may also occlude esophageal orifice leading to hindrance in eructation. This gives rise to dyspnea and death [33].

## Polybezoars

Hard stone-like masses that are formed by the deposition of salts around polythenes in digestive tract are known as polybezoars. These polybezoars not only cause hindrance in food passage but also leads to pain and inflammation of rumen [33].

## Traumatic Reticulo Pericarditis

Many times nails, wires, or other sharp hard objects are wrapped in poly bags and dropped in waste pits. While grazing on such waste, animals ingest these sharp objects along with plastic bags. Due to the honeycomb pattern of the reticulum, these sharp objects get trapped in reticulum causing damage. Over a period of time, these sharp objects penetrate the wall of the reticulum and diaphragm and invade heart leading to traumatic reticulopericarditis [33].

## Local or Diffused Peritonitis

Sometimes the animals accidentally ingest the sharp objects such as needles, nails, and wires wrapped in the plastic bags and covers. Over a period of time, these sharp materials are released in rumen causing ruminal wall puncture and development of local or diffused peritonitis [38,39].

## Poor Production

The physical presence of plastic foreign bodies in rumen and reticulum interferes with the absorption of volatile fatty acids in rumen and reticulum. Thereby, plastic foreign bodies may hamper the milk yield and the rate of animal fattening [19,35,38].

## Chemical Leaching in Rumen

In the manufacturing of polymeric materials, various additives are used depending on the type of produced polymers. These additives include plasticizers, antioxidants, catalysts, stabilizers, pigments, and fillers [40]. These additives are bound either chemically or physically into the polymers and may be present in their original or an altered form. Bonding between additives and polymers is not strong and is liable to be broken due to minor external influence [41]. In addition, the polymerization process may leave trace quantities of residual monomers of low molecular mass in the polymers.

Plastic waste materials present in the rumen are exposed to various destructive processes by physical and microbial actions. Physical action in the form of the churning of plastic present in rumen due to ruminal contractions and microbial action in the form of enzymatic activity by protozoa and other rumen microflora. These physical and microbial influences may cause the release of certain chemicals and monomers from the ingested plastic material into rumen fluid and then to circulation. Leaching of these chemicals may increase with the aging of accumulated plastic in rumen [22].

## Immunosuppression

In the manufacturing of plastics and polythenes, several chemicals such as bisphenols, polyvinyl chloride, cadmium, lead, and acrylamide are being used. These chemicals are known as immunosuppressants [33,42]. In animals accumulated with plastics in the rumen, there is the possibility of leaching of these chemicals from the rumen and causing immunosuppression.

## Heavy Metal Toxicity

Cobalt, lead, mercury, cadmium, chromium, and their salts and complexes such as stearates and phthalates are generally incorporated in the processing of plastics. These heavy metals leach out slowly from the rumen and reach circulation. Being cumulative poison, these toxic metals bioaccumulate in vital organs and cause harmful effects slowly over a period of time. The other threat of these heavy metals is their plausible presence in the human food chain through meat and milk products. Many studies have proven the presence of these heavy metals in stray animals grazing on waste, but their effect on host is not yet documented [43,44].

## Estrogenic Activity and Reproductive Problems

Plastic materials release many chemicals having estrogen activity such as bisphenol A (BPA), di-(2-ethylhexyl) phthalate (DEHP), and triphenyl phosphate (TPP) [45]. These xenobiotic chemicals often interact with more than one estrogen receptor subtypes and produce many biological and adverse health effects in mammals [46,47]. They may interfere with the reproductive processes of both males and females at several points of the reproductive cycle through a range of physiological mechanisms and cause cystic ovarian diseases, low spermatozoa count, early embryonic mortality, early puberty, etc. [47-49]. The physiological consequences are largely unknown for stray ruminants grazing on plastic waste materials. However, the levels of exposure to estrogenic hormones and phthalates in grazing ruminants are such that when studying fertility problems in high-yielding dairy cattle the impacts of exposure to these chemicals through the food and drinking water cannot be neglected [50].

## Carcinogenicity

Plastic waste materials accumulated in rumen over a period of time leach out chemicals such as polychlorinated biphenyls, dioxins, phthalates, and monomers [22]. These chemicals are highly reactive and biologically aggressive. Exposure to these chemicals over a period of time is known to cause various types of tumors such as hepatocellular carcinomas, testicular carcinomas, mammary gland tumors, and ovarian tumors in laboratory animals and human beings [49]. Studies indicating the occurrence of such conditions in ruminants harboring plastics are not available.

## Teratogenicity

Monomers and other chemicals released from these plastic materials in the rumen are biologically highly active [51]. They produce a teratogenic effect in newborns if their mothers are exposed to these chemicals during pregnancy [42]. Studies documenting such effects in ruminants are not available.

## Clinical Signs

Clinical signs noticed in the animals vary depending on the amount and duration of these foreign plastic materials ingested [23,33]. In the initial stages where animals have ingested small quantities of foreign plastic materials, they remain symptomless for months. Clinical signs are exhibited when the ingested plastic materials interfere with the normal functioning of rumen [11,17]. Depression, partial or complete anorexia, recurrent bloat, reduced milk yield, weight loss, suspended rumination, ruminal impaction, and increased susceptibility to other disease conditions are the most common symptoms observed in the affected animals [9,22]. As ruminal impaction due to plastic materials is primarily a disease of abandoned stray animals, and these animals often die due to road traffic accidents or slaughtering. Therefore, most of the time other clinical signs such as poor reproductive performance and tumors will go unnoticed and unreported [52].

## Diagnosis and Treatment

As clinical signs in animals suffering from ruminal impaction due to plastic materials are nonspecific, diagnosis of ruminal impaction due to plastic materials is a real challenge to clinicians. Recurrent bloat, persistent ruminal impaction, and history of grazing the animals along roadsides on garbage raise the suspicion of ruminal impaction due to plastic materials in animals [9,22]. Although few studies have attempted in detecting the chemicals leached from these plastic materials in ruminal fluid and milk samples, still much more work is required in using these chemicals for the diagnostic purpose [22]. Conservative approaches such as use of anti-bloat agents or purgatives are not successful in the management of plastic foreign body syndrome. Therefore, until today all physicians are dependent on rumenotomy for both diagnostic and therapeutic purposes of ruminal impaction due to plastic materials in animals [38].

## Impact on Human Health

Plastic materials accumulated in rumen release various chemicals such as plasticizers, monomers, BPA, DEHP, TPP, polychlorinated biphenyl’s, and heavy metals slowly over a period of time. Numerous workers have detected the presence of these chemicals in milk and meat of animals staying in urban areas [22,43,44,53]. These chemicals pose a great threat to human health by entering into the food chain through milk and meat products over a long period [53]. In human beings they may interfere with various cellular processes by acting as endocrine disruptors, carcinogens, teratogens, allergens, bioaccumulating agents, etc., causing adverse health effects in an intact organism, or its progeny, or (sub) population [54,55].

Endocrine disruptors are the exogenous substances that bind to nuclear hormone receptors and thereby act as an agonist or antagonist of the endocrine system [56]. Chemicals originated from plastic materials interfere with the functioning of various hormone receptors such as estrogen, thyroxin, and insulin and produce many health-related problems, such as early puberty in females, reduced sperm counts, altered functions of reproductive organs, cryptorchidism, altered sex-specific behaviors, increased rates of some breast, ovarian, testicular, and prostate cancers, obesity, hypothyroidism, and Type II diabetes [57-59]. Fetal, newborn and juvenile mammals, are especially sensitive to very low (sometimes picomolar to nanomolar) doses of endocrine disrupting chemicals and develop adverse and irreversible effects during development, thus putting a future generation in risk [58].

Unpolymerized monomers and other chemicals leached from plastic materials are highly mutagenic. Continuous exposure to such chemicals predisposes to the development of various tumors such as hepatocellular carcinoma, testicular carcinoma, breast, and ovarian tumors [56-58]. Exposure to such chemicals during uterine life causes the developmental defects. Other bioaccumulators, heavy metals such as cadmium and lead tend to accumulate slowly in the human body and produce harmful effects. Other effects such as altered learning abilities and aggressive behavior in human beings are linked to the exposure to such chemicals [45]. Till now there are no direct reports documenting these adverse effects from plastic foreign body impacted ruminants on human health.

## Prevention and Control Measures

Ruminal impaction due to plastic materials arises as a result of improper management of plastic waste and substandard animal husbandry practices [33,60]. Therefore, proper waste disposal practices and proper husbandry methods may be required to check environmental pollution and prevent animals from accessing indigestible foreign bodies [11,23]. Good animal husbandry practices can be followed by providing adequate feed, water, shelter, and mineral supplements timely. Establishing fodder banks, grazing centers and water facilities will help to mitigate the adverse effects of dry season by avoiding the straying of animals on roadsides and garbage in search of feed and water.

Plastic waste can be effectively managed by following four R’s, i.e., Reduction, Reuse, Recovery, and Recycling. For developing countries reduction in the generation of plastic waste and reuse of generated plastic waste are the feasible options [17]. Reduction in plastic usage can be achieved by usage of alternative materials, for example, Jute or cloth or paper bags instead of polybags. Municipality and other sanitation authorities should actively collect the plastic waste materials along roadsides and open grounds and dispose of them properly to avoid ingestion by animals. Awareness should be created among the public and in particular livestock owners about the harmful effects of plastic on animal health and, in turn, its effects on human health [61]. They should be educated about good animal husbandry practices and disposal of plastic waste.

## Conclusion

In today’s era plastic materials have become inseparable parts of our life. Due to the low cost, easy availability, strength, moisture resistance, flexibility, and many other characteristics, and plastic materials have widespread applications and are omnipresent. It is not the use of plastic itself, but it is their improper waste disposal that is creating detrimental effects on human, animal, and environmental health. Ruminal impaction due to plastic materials is one of the major but often neglected health issues of stray ruminants in urban areas of developing countries. Scientific literature is in the form of case reports or slaughterhouse incidence. Much more basic research is warranted to explore the pathophysiology, diagnosis, and treatment of ruminal impaction due to plastic materials in animals. Although few workers have reported the presence of plastic residues in milk and meat products of animals suffering from ruminal impaction due to plastic materials. Detailed investigations in the directions of chemical leaching in the rumen are required. Apart from improving basic animal husbandry practices and disposal of plastic waste, intervention by government particularly on slaughter policies of unproductive animals, establishment of fodder banks/grazing centers/watering facilities for animals, starting public health awareness programs, implementing strict laws for proper disposal of plastic waste, etc., will help in controlling ruminal impaction due to plastic materials in animals.

## Authors’ Contributions

MP conceptualized the review, collected the literature and prepared the manuscript. SD studied and edited the manuscript. Both authors read, finalized, and approved the manuscript.
